# Clinical value and advances of electromyography in cervical dystonia: a review

**DOI:** 10.3389/fneur.2026.1759508

**Published:** 2026-04-10

**Authors:** Muqier Bao, Di Ma, Mengyi Zhou, Xiaohui Ma, Huaiyu Sun, Lixia Zhu, Shuai Hou, Hongmei Meng

**Affiliations:** Department of Neurology, The First Hospital of Jilin University, Changchun, China

**Keywords:** botulinum toxin, cervical dystonia, deep brain stimulation, diagnosis, differential diagnosis, efficacy evaluation, electromyography, pathophysiological mechanism

## Abstract

Electromyography (EMG) is a definitive diagnostic tool that has become indispensable in the clinical management of cervical dystonia (CD). This review systematically delineates its pivotal role across the entire care pathway. EMG provides crucial electrophysiological evidence for the diagnosis and differential diagnosis of CD by identifying abnormal co-contractions and “recruitment” phenomena. In therapeutic management, EMG-guided botulinum toxin injections enable precise targeting of affected muscles, significantly improving therapeutic efficacy and reducing adverse reactions. It also offers objective references for target muscle selection and parameter optimization in interventions such as deep brain stimulation. Research on pathophysiological mechanisms reveals that EMG captures abnormalities in central pattern generators, offering insights into the underlying neurophysiological mechanisms of CD. Moreover, EMG serves as a reliable tool for quantifying therapeutic effects and conducting long-term follow-ups. Despite limitations such as operator dependence, emerging technologies—including high-density surface EMG and machine learning—are expanding the role of EMG in precision medicine and individualized treatment strategies for CD. This review highlights the clinical value, advances, and future prospects of EMG in the diagnosis, classification, pathophysiological research, treatment guidance, and efficacy evaluation of CD.

## 1 Introduction

Cervical dystonia (CD) is the most common form of focal dystonia, characterized by involuntary muscle contractions that cause abnormal movements and postures of the head, neck, and shoulders (1). It is also the most common form of primary dystonia (2). Although the specific pathogenesis of CD has not been fully elucidated, existing studies suggest that it may be associated with abnormalities in brain structures such as the basal ganglia, thalamus, and cerebellum, as well as the dysfunctional neural network connections among these regions. Its occurrence is influenced by multiple factors (3). The global prevalence rate of CD is approximately 5–9 per 100,000 individuals. Although classified as a rare disorder, its high disability rate and chronic course result in persistent challenges such as abnormal head and neck postures, pain, and social dysfunction. Approximately 70% of patients are unable to drive, work, or eat normally due to forced head tilting, and nearly 40% experience comorbid depression or anxiety (4). Currently, local injection of botulinum toxin type A is considered the first-line treatment for CD (5). Although botulinum toxin type A demonstrates significant efficacy, a considerable proportion of patients report adverse outcomes and eventually discontinue treatment (6).

The EMG is a fundamental neuroelectrophysiological diagnostic procedure that provides a direct window into the function of the “final common pathway” from the spinal anterior horn cells to the muscles by recording their electrical activity (7, 8). Its essential role in clinical neurology lies in localizing lesions (e.g., differentiating radicular, plexus, or peripheral nerve injuries), distinguishing neurogenic from myopathic disorders, and quantifying muscle activation and recruitment patterns (9–12). In the field of movement disorders, EMG analysis extends beyond simple lesion detection to precisely capture the temporal dynamics of abnormal movements, intermuscular co-contraction, and pathological oscillatory rhythms, thereby offering irreplaceable objective insights into the physiological mechanisms underlying clinical symptoms (13–16).

Building upon this foundation, EMG has established itself as an indispensable tool in the contemporary management of CD. Beyond its well-documented role in enhancing the precision and outcomes of botulinum toxin therapy (13, 14, 17–19), EMG has also been used to provide critical electrophysiological diagnostic evidence (20). Importantly, advanced signal analysis techniques—such as coherence and spectral analysis—capture the distinct cortico-subcortical oscillatory signature of CD, characterized by a loss of physiologic rhythms and the emergence of pathological low-frequency synchronization (21). These signatures offer direct, objective insights into the underlying circuit-level dysfunction, bridging peripheral symptoms with central network pathophysiology (22–25). Accordingly, this review aims to systematically synthesize the multifaceted applications of EMG across the entire spectrum of CD care, detailing its utility in: (1) elucidating circuit-level pathophysiology; (2) diagnosis and phenotypic characterization; (3) guiding targeted interventions (botulinum toxin and deep brain stimulation); and (4) providing objective metrics for treatment evaluation and long-term monitoring.

## 2 Search strategy

This narrative review was conducted through a systematic search of English-language literature up to November 2025. Electronic databases included PubMed, Web of Science, Embase, and the Cochrane Library. Search terms combined keywords and controlled vocabulary (e.g., MeSH, Emtree) such as: “cervical dystonia,” “spasmodic torticollis,” “electromyography,” “EMG,” “botulinum toxin,” “deep brain stimulation,” “pathophysiology,” “diagnosis,” and “treatment.” No start date restriction was applied. The reference lists of retrieved articles were manually screened for additional relevant sources. Literature selection was based on relevance to the clinical, pathophysiological, or therapeutic aspects of EMG in CD, prioritizing original studies and authoritative reviews. Case reports and studies with major methodological limitations were excluded.

## 3 Pathophysiological mechanisms of CD and insights from EMG

### 3.1 Introduction: EMG as a window into central motor pathophysiology

The symptoms of CD originate from dysfunctional communication within central motor control networks. EMG provides a unique and direct *in vivo* window into these pathophysiological processes by translating aberrant neural commands into recordable muscular electrical activity.

### 3.2 Electrophysiological basis of abnormal motor patterns

The EMG serves as a pivotal tool for elucidating the pathophysiology of CD by translating aberrant central motor commands into decipherable muscular signals. A hallmark of this central dysfunction is the excessive, pathological co-contraction of agonist–antagonist muscle pairs and abnormal synchronization across synergistic muscles, driven by impaired central inhibitory control (26).

The EMG recordings in patients with CD consistently reveal characteristic signatures of this disinhibition. These include: (1) persistent or intermittent discharges that maintain abnormal postures, indicating a failure to suppress unwanted muscle activity; (2) high-amplitude potentials often exceeding 50% of maximal voluntary contraction, reflecting exaggerated motor unit recruitment; (3) spontaneous low-to-moderate frequency (4–9 Hz) tremor-like activity, suggesting oscillatory network instability; and (4) paradoxical co-activation of antagonistic muscles during voluntary movement, a direct sign of lost reciprocal inhibition. Notably, these abnormal activities are often modifiable by sensory tricks, highlighting the role of sensorimotor integration in their generation (13, 14).

Collectively, these EMG features provide direct electrophysiological evidence for the loss of normal inhibitory regulation within the corticobasal ganglia-thalamic circuits in CD (27).

### 3.3 Alterations in rhythmic oscillatory activities and their central origins

#### 3.3.1 Loss of physiological rhythms and their implications

In healthy individuals, precise motor control is supported by distinct, task-dependent oscillatory rhythms within the central nervous system, which are reflected by EMG. Notably, similar 10–15-Hz oscillations observed in the deep and superficial neck muscles indicate that cervical motor neurons are activated by common central drives. This is in sharp contrast to limb muscles, which typically exhibit higher-frequency oscillations (15–35 Hz) associated with direct corticospinal drive. The reticular formation, with its broad projections to cervical motor neuron pools, has been proposed as a major source of this common oscillatory drive for the neck. This indirect pathway is further supported by the findings of Baker et al., who showed that during stable contractions, cortical local field potentials (LFP) phase-lock with pyramidal tract neuron oscillations, and coherence between these LFPs and limb muscle EMG is observed in the 20–30-Hz band. These observations collectively suggest that voluntary cortical control of neck muscles involves a relay through the reticular formation, rather than relying solely on direct corticospinal pathways (15, 28–30). Concurrently, synchronization between muscles and the contralateral sensory-motor cortex at 15–30 Hz is also observed during voluntary contractions, representing another layer of physiological oscillatory coupling (31, 32).

In patients with dystonia, the characteristic 8–14-Hz self-spectrum peaks typically observed in healthy individuals are absent during isometric movements. This “peak” can be understood as a distinct, rhythmically recurring signal intensity maximum in muscle electrical activity. It functions akin to a healthy physiological metronome, stabilizing and coordinating muscle contractions. If these physiological peaks are indeed generated by oscillations within the reticular formation, their disappearance provides electrophysiological support for the involvement of the reticulospinal pathway in dystonia pathophysiology. Instead of these normal rhythms, a distinct pathological pattern emerges: the EMG activity in dystonia is frequently characterized by prominent oscillations in the 4–7 Hz range. This aberrant low-frequency drive is considered a hallmark of dystonia and is thought to originate from dysfunctional oscillatory activity within the basal ganglia (13, 24, 25). The shift from the absent physiological peaks to this prominent pathological rhythm underscores a fundamental alteration in central oscillatory dynamics in CD (16, 23, 33).

The loss of the physiological 8–14 Hz spectral peak constitutes a key electrophysiological signature in CD. Consistent evidence indicates that this peak, typically prominent in the splenius capitis (SPL) muscle of healthy individuals, is generally attenuated or absent in the affected muscles of patients. This phenomenon extends beyond the SPL; a similar trend toward the diminution of this peak, frequently accompanied by an increase in autospectral power within the 3–10 Hz band, has been observed in other cervical muscles (e.g., the semispinalis capitis), suggesting a shift of oscillatory energy to lower frequencies as a common pathological pattern across neck muscle groups (22, 24, 26). While this finding has significant diagnostic implications, its application is subject to specific limitations. First, the absence of the peak is not uniformly present across all affected muscles, and its discriminatory power is influenced by muscular state. For instance, in the SCM, this characteristic peak may be detectable only during active muscle contraction (16). Second, even in healthy controls, the expression of this physiological peak can be variable due to several factors: (1) in specific isometric tasks, a muscle may not be recruited into the synergy promoted by this rhythm; (2) inherent differences in neural innervation between muscles (e.g., cranially innervated SCM vs. dorsally innervated muscles) may influence their oscillatory properties (22); and (3) in non-restrained postures, reflexive feedback triggered by dystonic and/or tremor movements may mask or mimic normal rhythmic activity, complicating result interpretation (23).

#### 3.3.2 Emergence of pathological low-frequency drive

Previous studies have established that analyzing intermuscular coherence across frequency bands holds diagnostic value for identifying pathological synergistic contraction between muscles. Intermuscular coherence is a quantitative measure that assesses whether the electrical signals from different muscles are synchronized at specific frequency “beats.” High coherence indicates that these muscles are stepping in coordinated unison at a particular rhythm. In patients with dystonia, EMG recordings consistently exhibit coherence characteristics within the low-frequency band, primarily concentrated between 4 and 7 Hz (16, 33). Notably, such a “dystonic drive” pattern is generally absent in healthy controls, as several studies have failed to identify comparable coherence in this frequency range (34–36). This low-frequency coherence tends to be particularly pronounced in patients with marked rhythmic or periodic activity, such as tremor or dystonic movements, reflecting an enhanced thalamocortical drive subsequent to disinhibition of the basal ganglia (27, 34, 35, 37, 38).

Healthy individuals may also exhibit coherence in other frequency bands. For instance, Tijssen et al. reported that during autonomous, continuous neck muscle contractions, healthy controls displayed significant coherence in the 0.5–1.5 Hz and 8.1–15.1 Hz bands, with a distinct coherence peak at 10–14 Hz—a feature that was notably reduced in patients with CD (16). Conversely, other studies have indicated that the low-frequency coherence observed in the neck muscles of healthy controls can be similar in magnitude to that seen in patients with CD (16, 23, 39). Adding to this complexity, van Rootselaar et al. (39) described a low yet significant 4–8 Hz coherence between hand and arm muscles in healthy individuals during posture maintenance tasks. Although statistical comparisons may not always reveal a significant difference in low-frequency coherence between CD patients and healthy controls, patients consistently exhibit higher coherence levels (23).

Cross-correlation analysis offers further insight into pathological synchronization. In mammals, a peak with a duration of 6 ms near time zero in cross-correlation plots indicates short-term synchronization of motor units (40). The presence of a broader peak around 40 ms (wide-peak synchronization) suggests a common presynaptic input to the motor neurons (41). Farmer et al. highlighted a neurophysiological distinction between pathological co-contraction in dystonia and voluntary synergistic contraction. In patients with upper limb dystonia, cross-correlation analysis of antagonistic muscles (wrist flexors and extensors) revealed a peak at time zero lasting 35 ms, indicating synchronous motor unit activity with wide-peak characteristics. In contrast, cross-correlation plots from healthy individuals showed no evidence of such motor unit synchronization (42). That is, the cross-correlation histograms of wrist flexor and extensor EMG in controls appeared flat. Even when healthy participants attempted to mimic dystonia by rapidly co-contracting muscles to produce a simulated 200 ms cross-correlation peak, the resulting pattern remained markedly different from that observed in dystonia patients.

Despite its high specificity (98%), targeting the 4–7 Hz “dystonic” drive has very low sensitivity (17%) for detecting dystonic muscles (25), underscoring the need for complementary diagnostic approaches (33).

#### 3.3.3 Corticomuscular coherence in circuit dysfunction

Research confirms that a core deficit in the motor control of patients with dystonia lies in the impaired dynamic regulation of sensorimotor cortical oscillations. Studies utilizing high-density electroencephalography reveal a generalized reduction in task-related α- and β-band desynchronization in patients, indicating the cortex’s inability to flexibly switch its rhythmic state according to motor demands (21). This “failure” in cortical rhythm switching likely leads to the transmission of aberrant, rigid oscillatory commands downstream to the motor network. These abnormal commands originating from the central nervous system are manifested in the synchronized activity of peripheral muscles, such as the 4–7 Hz intermuscular drive. Corticomuscular coherence (CMC) analysis—achieved by synchronizing EEG/MEG recordings with surface electromyography—directly quantifies this central-peripheral coupling. It is noteworthy that this EMG-based CMC method captures not only cortical oscillations but also reveals the oscillatory integration between the cortex and subcortical nuclei, providing a direct electrophysiological window into the circuit-level dysfunction underlying CD symptoms.

In electrophysiological studies of the pathophysiological mechanisms of dystonia, a fundamental distinction exists in corticomuscular coupling patterns between healthy and diseased states. In healthy individuals during voluntary movement, coupling between the sensorimotor cortex and contralateral muscles is primarily characterized by enhanced physiological *β*-band coherence (43, 44). A pattern reflecting normal cortical drive during motor preparation and execution. However, in patients with dystonia, this physiological pattern is significantly disrupted and replaced by pathological hypersynchronization concentrated within the low-frequency range of 4–12 Hz (45, 46). Such abnormal CMC is merely the external manifestation of broader circuit dysfunction. Local field potential recordings confirm that the same pathological low-frequency oscillations are also markedly enhanced in key subcortical structures, including the globus pallidus internus (GPi) and the subthalamic nucleus. Notably, oscillatory activity originating from the GPi demonstrates directional drive toward affected muscles, underscoring its role as a central “pacemaker” within the aberrant network (37, 47, 48). The causal significance of this stable pathological low-frequency synchronization network is further validated by its specific responses to interventions at different levels. On one hand, central deep brain stimulation can effectively and reversibly suppress the power of low-frequency oscillations recorded from the GPi and their coherence with cortical/muscular activity (49, 50). On the other hand, peripheral effective sensory tricks can induce a shift from synchronization to desynchronization in the 4–8 Hz band of oscillatory activity between the sensorimotor cortex and the globus pallidus (51). These two distinct intervention modalities alleviate symptoms by modulating the same pathological oscillatory network, strongly confirming that abnormal low-frequency synchronization is a core network feature driving dystonic symptoms and can be precisely regulated.

Peripheral sensory input can modulate the activity of neural oscillations (51, 52). A key example is that during the performance of a sensory trick in patients with cervical dystonia, dynamic modulation of pathological low-frequency synchronized oscillations in the globus pallidus occurs, characterized by a reduction in oscillatory power. This real-time sensorimotor integration may transiently disrupt the pathological circuit activity driving muscle contractions (53). This observation highlights that neural oscillatory patterns are not static but are dynamically influenced by sensorimotor state and task demands. Across the corticobasal ganglia-thalamus network, oscillatory patterns vary with different phases of motor tasks. For instance, β-band activity (13–30 Hz) is most prominent during posture maintenance but typically decreases during movement initiation and is replaced by γ-band rhythms (>30 Hz) during action execution (45, 54–60). This normal, task-dependent modulation of oscillations stands in stark contrast to the rigid, pathological low-frequency synchronization that characterizes CD.

## 4 Clinical applications and benefits of EMG in CD management

### 4.1 Diagnostic and differential diagnostic value

The diagnosis of CD, while primarily clinical, can be challenging due to phenotypic heterogeneity and the existence of mimics. Reliance solely on clinical presentation (e.g., type of head deviation), physical signs (e.g., muscle hypertrophy), or ancillary imaging such as cervical spine MRI and musculoskeletal ultrasound, is insufficient for achieving a definitive diagnosis or precise phenotypic classification. These modalities cannot directly capture the dynamic, involuntary muscle activity that is the hallmark of dystonia. In this context, EMG emerges as a critical and often decisive tool, providing unique electrophysiological evidence that is unobtainable by other means. This approach necessitates determining whether secondary dystonia is present and ruling out pseudodystonia (17). Although clinical assessments (including the detection of abnormal postures, movement patterns, involuntary tremors, muscle hypertrophy, and the often pathognomonic presence of sensory tricks) form the cornerstone of diagnosis (61), they must be supplemented by detailed history, comprehensive examination, and targeted investigations to exclude secondary causes such as multiple sclerosis, spinocerebellar ataxia, systemic juvenile idiopathic arthritis, cervical spine lesions, or early Parkinson’s disease (62–65).

Within this diagnostic framework, EMG serves as a critical and decisive tool. The challenge of differentiating true dystonia from pseudodystonia—a broad category of conditions mimicking dystonia through peripheral, spinal, brainstem, thalamic, cortical, or non-neurological mechanisms (66)—highlights the need for objective physiological evidence, which can be obtained using EMG.

The definitive diagnostic power of EMG is most clearly demonstrated in its ability to objectively distinguish CD from other hyperkinetic movement disorders based on precise electrophysiological signatures. Although the EMG characteristics of CD are distinctive, differentiation from other common movement disorders—such as tremor, myoclonus, tic disorder, and chorea—remains necessary in clinical practice. EMG provides crucial objective evidence through the analysis of discharge patterns, rhythm, synchrony, and responses to specific physiological examinations. For example, tremors (including physiological tremors, tremors in Parkinson’s disease, and idiopathic tremors) show rhythmic 4–12-Hz fixed-frequency discharges without abnormal background EMG activity or fixed posturing (67, 68). Tremors associated with dystonia tend to be weaker and more irregular in terms of amplitude and frequency (20). The duration of myoclonic discharges is relatively short, generally not exceeding 100 ms and exceeding 250 ms in very rare cases, and these discharges show irregular EMG activity with a wide range of frequency distribution (69). Tic disorders are characterized by repetitive and stereotyped muscle spasms, with EMG activity returning to the resting state between spasms; they are also associated with premonitory sensations and are temporarily controllable by consciousness (70, 71). Chorea displays random EMG activity without a pattern, manifesting as diffuse, migratory, and irregular bursts (72). The distinctive EMG profiles of CD compared to those of hyperkinetic disorders are summarized in Table 1. These clear, reproducible electrophysiological boundaries allow EMG to be a source of decisive evidence for the differential diagnosis of CD.

**Table 1:** Electromyographic characteristics of cervical dystonia and common mimics for differential diagnosis.

Disorder	Key EMG patterns and signatures	Distinguishing features from CD
Cervical dystonia (CD)	Persistent/Intermittent Discharges: Involuntary muscle activity that sustains abnormal postures. Paradoxical Co-activation: Synchronous contraction of antagonistic muscles during voluntary movement when they should normally relax. Abnormal Recruitment: High-amplitude potentials often exceeding 50% of maximal voluntary contraction, indicating exaggerated motor unit recruitment. Modifiability by Sensory Tricks: The above abnormal activities can be temporarily altered or suppressed by specific sensory stimuli (e.g., touch). Supporting Evidence: Often accompanied by characteristic enhancement of 4–7 Hz intermuscular coherence and attenuation of the physiological 8–14 Hz oscillatory peak.	Reference standard. This column defines the comparative basis for other disorders.
Tremor disorders (e.g., Essential Tremor, Parkinsonian Tremor)	Rhythmic, oscillatory bursts within a relatively fixed frequency range (typically 4–12 Hz). Lack of sustained, abnormal background EMG activity between tremor bursts. Absence of fixed dystonic posturing driven by continuous muscle activity.	Lacks the sustained co-contraction and posturing of CD. Absence of the specific 4–7 Hz “dystonic” coherence pattern. Tremor bursts are more regular in amplitude and frequency compared to dystonic tremor.
Myoclonus	Very short-duration discharges (usually <100 ms, rarely up to 250 ms). Irregular EMG activity with a wide frequency distribution.	Discharge duration is markedly shorter than the sustained/tonic activity in CD. Pattern is irregular and asynchronous, lacking the organized co-contraction or specific oscillatory drive of CD.
Tic disorders	Stereotyped muscle spasms. EMG activity returns to baseline (resting state) between spasms. Often associated with premonitory sensations and temporary voluntary suppressibility.	Activity is intermittent and discrete, unlike the persistent or posture-sustaining activity in CD. Presence of a premonitory urge and voluntary suppressibility, which are atypical for CD.
Chorea	Completely random, non-patterned EMG activity. Manifests as diffuse, migratory, and irregular bursts of muscle activation.	Lacks any form of sustained synchronization, oscillation, or patterning (e.g., co-contraction, coherence) seen in CD. Movements are random and flowing, in contrast to the patterned, often directional posturing in CD.
Pseudodystonia	EMG findings are highly variable and depend on the underlying etiology (e.g., orthopedic, structural, psychogenic). A key feature is the absence of the specific electrophysiological signatures of CD listed above.	Absence of characteristic CD markers (e.g., specific coherence patterns, loss of 8–14 Hz peak). Diagnosis relies heavily on excluding primary neurogenic patterns and identifying an alternative cause through clinical history, exam, and imaging.

CD, cervical dystonia; EMG, electromyography; Hz, hertz.

### 4.2 Guidance for botulinum toxin therapy

#### 4.2.1 Challenges associated with precision botulinum toxin therapy for CD

Currently, local intramuscular injection of botulinum neurotoxin is the first-line treatment for CD. However, approximately 30–40% of patients still experience poor outcomes, primarily due to inaccurate targeting of affected muscles or suboptimal distribution of the neurotoxin dose (6).

This therapeutic challenge stems from the intrinsic complexity of CD. Patients exhibit heterogeneous abnormal postures and movement patterns owing to variations in muscle activation. The overlapping anatomical and functional roles of multiple neck muscles, which often span several joints, mean that even similar abnormal postures can result from significantly different patterns of muscle involvement. Furthermore, key dystonic muscles are frequently located in deep layers, evading reliable detection by routine physical examination and palpation. Compounding this difficulty, muscle activation patterns can change during treatment, and the development of compensatory muscle activity further complicates the accurate selection of injection targets (73). In current practice, the selection of muscles for botulinum toxin injection relies largely on clinical features—including abnormal posture, palpable muscle hypertrophy, and pain (74, 75)—which, while essential, may lack the precision required for consistently optimal outcomes.

#### 4.2.2 EMG as a guidance tool: from identification to quantification

##### 4.2.2.1 Identification and localization of dystonic muscles

EMG serves as a pivotal auxiliary technique for guiding botulinum toxin injections by providing objective identification and localization of dystonic muscles with high sensitivity and specificity. Its primary utility lies in overcoming key clinical limitations: EMG can detect and monitor activity in deep, non-superficial muscles inaccessible to routine examination. Crucially, it can verify the presence of characteristic abnormal discharges—such as sustained (tetanic) or tremor-like activity—at rest or during specific tasks. This capability enables the critical distinction between primarily active (dystonic) muscles and secondary compensatory muscles, refining target selection beyond clinical assessment alone (76, 77). Practical applications include acoustic feedback–based EMG devices, which can assist in real-time localization of hyperactive muscles during injection procedures.

##### 4.2.2.2 Quantitative assessment for dosage guidance

The role of EMG extends to quantitative analysis, which further refines localization and provides a crucial reference for determining the injection dosage. Quantitative EMG analysis includes the assessment of the number of turns per second and the mean amplitude of motor unit action potentials, which serve as indicators of involuntary muscle activity in CD. Previous research has reported the relationship between these parameters and muscle force: the number of turns and amplitude initially increase as force gradually increases, with average amplitude linearly correlated with force. However, the number of turns near maximum force significantly decreases, a phenomenon potentially influenced by factors such as action potential changes and muscle fatigue. Comparing these EMG patterns with those of healthy individuals under similar conditions provides valuable support for both muscle localization and dosage guidance (78).

In clinical practice, specific quantitative thresholds can indicate pathological hyperactivity. For example, a frequency of muscle contractions exceeding 100 times per second during attempted rest or amplitudes greater than 100 μV have been associated with abnormal neck muscle activity (18, 79–81). The clinical impact of this quantitative guidance is significant. Werdelin et al. (82) demonstrated that, compared to treatment based solely on clinical judgment, EMG-guided therapy (using a threshold of >100 contractions per second to define dystonic muscles) resulted in a greater reduction in dystonic activity, required smaller doses of botulinum toxin, and involved fewer injections into non-dystonic muscles. This is particularly relevant given the relatively low sensitivity (0.35) and specificity (0.74) of physical examination alone for detecting affected muscles. To further optimize identification, De Bruijn et al. (25) evaluated methods such as “minimum EMG” (activity during rest or the second-maximal voluntary contraction) and “overall mean EMG” (average activity across all contraction directions). This approach accurately predicted 54% of clinically diagnosed dystonic muscles and 91% of non-dystonic muscles, representing a refined strategy for target selection (25).

It is essential to integrate these electrophysiological findings with anatomical and physiological knowledge. The physiological characteristics and sensitivity to botulinum toxin vary across muscle groups. For instance, posterior neck muscles may require a higher injection dose than the sternocleidomastoid muscle to achieve the desired therapeutic effect. Therefore, the final determination of injection dose should be guided not only by quantitative EMG findings but also by relevant physiological differences among target muscles.

#### 4.2.3 Optimizing injection technique: targeting the motor endplate zone

Following the precise identification of dystonic muscles, optimizing the intramuscular injection site is critical for maximizing therapeutic efficacy and efficiency. Targeting the motor endplate zone (MEZ), the region with the highest density of neuromuscular junctions, is a key strategy for achieving a potent clinical effect while minimizing the required botulinum toxin dose (83, 84).

High-density surface EMG (HD-sEMG) has emerged as a valuable, non-invasive tool for accurately mapping the MEZ of cervical muscles (84–86). This technique localizes the MEZ by detecting characteristic phase inversion patterns in the electrical signal when electrode arrays are aligned along the muscle fiber direction (87). The clinical utility of this precision is significant: HD-sEMG-guided injection into the MEZ can produce a therapeutic effect equivalent to that of a standard full-dose injection using only half the dose, thereby enhancing treatment efficiency and potentially reducing side effects (83, 84).

Importantly, HD-sEMG studies have provided precise anatomical guidance for common injection targets. For instance, research has confirmed that the MEZ is typically located at the lower border of the upper third of the SCM muscle, while in the SPL, it is generally situated in the mid-portion of the muscle (88). This electrophysiologically-defined anatomy offers a valuable practical reference for clinical injections.

### 4.3 Informing surgical interventions and deep brain stimulation

Preoperatively, EMG holds strategic value in the surgical planning of deep brain stimulation (DBS) for CD, extending its utility to the guidance of central target selection. Pathophysiological studies, such as corticomuscular coherence analysis, have implicated dysfunction within the corticobasal ganglia-thalamic network in CD. A pivotal study by Chen et al. provides direct electrophysiological evidence demonstrating that pathological low-frequency oscillatory activity (3–12 Hz) is markedly enhanced and highly synchronized within the GPi of dystonia patients. This finding offers a key pathophysiological explanation for why the GPi has emerged as a primary target for DBS intervention. Building upon this foundation, meticulous analysis of abnormal cervical muscle activity patterns, such as specific synergistic co-contractions and tremor characteristics, can provide critical physiological rationale for the individualized selection of either the GPi or the subthalamic nucleus (STN) as the surgical target (51, 89).

Intraoperative functional physiological confirmation of the DBS target can be achieved by analyzing the coherence between LFPs recorded from the tentative target (e.g., GPi) and surface EMG from affected muscles. The site exhibiting maximal LFP-EMG coherence within the pathological frequency band (e.g., 4–12 Hz) represents the symptomatic “hotspot,” providing real-time, circuit-based evidence to complement anatomical targeting. Concurrently, motor evoked potential (MEP) threshold testing serves as a key method for assessing the proximity of the DBS electrode to the internal capsule (IC). This technique does not rely on patient feedback, making it particularly useful for surgeries under general anesthesia. Research indicates that MEP thresholds can effectively gauge electrode proximity to the IC, and this method has been applied and validated for targeting structures including the STN, ventral intermediate nucleus (VIM), and GPi (90, 91). Earlier studies corroborate this finding, demonstrating a correlation between MEP-determined IC proximity and improved accuracy in final electrode placement (92, 93).

Following implantation, EMG provides objective physiological guidance for the long-term management of DBS therapy. During initial and follow-up programming sessions, surface EMG can serve as a real-time biofeedback tool to assess stimulation efficacy. By simultaneously recording EMG activity from target muscles, clinicians can directly observe the immediate suppressive effects of different parameter combinations (voltage, frequency, pulse width) on abnormal muscle discharges (e.g., tonic or tremulous bursts), thereby aiding in the determination of the optimal settings. Furthermore, quantitative EMG data (e.g., mean amplitude, burst frequency, or co-contraction index of abnormal activity) can serve as an objective biomarker for long-term therapeutic efficacy. Compared to reliance on subjective clinical scales alone, the quantitative data provided by EMG can more sensitively reflect microscopic changes in symptoms, informing adjustments to the therapeutic strategy. This approach also lays the groundwork for the future development of adaptive DBS systems based on electrophysiological signals. Furthermore, the quantitative recording of muscle response characteristics by EMG provides objective metrics for optimizing postoperative individualized stimulation parameters (94, 95).

### 4.4 Quantitative evaluation of treatment efficacy and long-term follow-up

Following botulinum toxin injection, functional denervation of muscle fibers occurs due to the toxin’s blockade of presynaptic acetylcholine release. This mechanism results in characteristic changes in the EMG data of neck muscles at the injection site (96). Accordingly, comparing the changes in EMG data before and after injection allows for the objective detection and evaluation of the efficacy of botulinum toxin injection. A double-blind, placebo-controlled study conducted by Ostergaard et al. (18) showed that, following botulinum toxin injection in patients with CD, the number of revolutions per second and the average amplitude of the injected muscles decreased, whereas the ratio remained relatively stable. This indicates a reduction in the number of motor units involved in involuntary activities and/or a decrease in their discharge rate, ultimately resulting in diminished involuntary muscle activity. Based on these findings, the decrease in the frequency of involuntary contractions after botulinum toxin injection appears to be a reliable indicator of therapeutic benefit (18).

Brans et al. further demonstrated that changes in EMG activity induced by botulinum toxin injection can serve as a reliable physiological indicator of treatment response. They found that the overall reductions in EMG activity were significantly associated with the improvements in clinical indicators, including EMG amplitude and the Tsui scale, and the TWSTRS-Disability score (79). These changes typically emerge approximately 6 weeks after injection and gradually return to pre-treatment levels by around 30 weeks. This may reflect the reversible, random loss of muscle fibers caused by presynaptic denervation (97). However, with repeated botulinum toxin injections—approximately seven times—such changes may gradually become less significant, and delayed similar changes may occur in the unaffected, non-tremor muscles on the opposite side (98). Owing to physiological differences among individuals and the varying characteristics of different neck muscles, injecting a fixed botulinum toxin dose may produce different magnitudes of changes in EMG parameters such as the number of turns, amplitude, and ratios (99). This underscores the need for evaluating the therapeutic effects of different muscles, as well as differentiated assessment criteria to ensure the accuracy of assessment results. The multifaceted applications and core value of EMG across the entire spectrum of CD diagnosis, treatment, and research are comprehensively summarized in Table 2. In addition to analyzing the number of turns and amplitude of EMG, several other methods can be used to quantify the effects of botulinum toxin injection. For example, evaluating the functional denervation phenomenon of individual muscle fibers caused by the action of botulinum toxin and detecting the fibrillation potential characteristics can be used as important indicators of therapeutic efficacy.

**Table 2:** Summary of the core values of EMG in cervical dystonia research and clinical care.

Application/research field	Core EMG indicators and methods	Research value and significance
Target muscle identification, disease diagnosis, and differential diagnosis	Surface or needle EMG: Identify the abnormal burst potentials and the phenomenon of strong, high-frequency discharges	Objective identification of overly active individual target muscles (such as SCM, SPL, and SESP) provides direct evidence for diagnosis and differential diagnosis, avoiding subjective judgment errors.
Pathological pattern recognition	Power spectrum analysis: As the spectral peak disappeared and the low-frequency shift occurred	Some muscles of patients with CD reflecting the fatigue state of muscles and abnormal patterns of neuronal discharges. These are the characteristic manifestations at the muscle level of central drive alterations (such as the disappearance of 8–14-Hz self-spectrum peaks).
Exploration of the central mechanism	Intermuscular coherence and cross-correlation diagram	The identification of abnormal central synchronization drive as the root cause of pathological synchronous contractions in multiple muscles provides electrophysiological evidence for the central generator hypothesis (such as detecting the synchronous 4–7-Hz low-frequency drive in dystonic muscles and the appearance of wide cross-correlation peaks).
Treatment guidance and quantification	Minimum and total average EMG Number of turns and amplitude	Achieving precise selection and individualized application of botulinum toxin injections: Selection of injection targets precisely based on electrical activity and quantification to guide the injection dosage. This enhances dosage guidance, improves the therapeutic effect, and reduces side effects (such as abnormal activity of neck muscles, which may occur during rest, with a transition frequency greater than 100 times per second or an amplitude greater than 100 μV).
Optimization of injection techniques	High-density surface EMG	Non-invasive and precise location of the muscle endplate area guide botulinum toxin injection to the densest area of the neuromuscular junction, thereby maximizing drug efficacy and reducing the total dose (for example, in the sternocleidomastoid muscle, the MEZ is located at the lower border of the upper one-third part of the muscle).
Neural circuit localization	Cortical muscle coherence Analysis of frequency band oscillations in the exercise task (such as the β band)	Identify the cortical sources of abnormal oscillations (such as the sensory-motor cortex), deepen the understanding of the pathophysiological mechanisms, and provide neurophysiological basis for the selection of target points and parameter regulation in DBS surgery.
Objective evaluation of the therapeutic effect	Comparison before and after treatment: number of turns, amplitude, synergy contraction index, and denervated potential	Provide quantitative and objective efficacy evaluation indicators, which are superior to simple clinical scales. They can be used for long-term follow-up to assess the progression of the disease and the necessity of adjusting the treatment plan.

SPL, splenius capitis; SCM, sternocleidomastoid muscle; SESP, semispinalis capitis muscle; EMG, electromyography; MEZ, motor endplate zone; DBS, deep brain stimulation surgery.

## 5 Current limitations and future directions

Despite providing crucial objective physiological information for the diagnosis and treatment of dystonia, EMG still exhibits multiple limitations that may affect its diagnostic specificity, standardization, and clinical accessibility. In terms of diagnosis, the absence of unified and quantitative criteria for identifying abnormal discharges restricts its diagnostic specificity, and it remains challenging to distinguish dystonia from other movement disorders based solely on EMG patterns. Regarding therapeutic guidance, current techniques are insufficient for accurately and non-invasively differentiating primary dystonic muscles from secondary compensatory ones, particularly in detecting deep muscle activity. While EMG can localize muscle activation, it cannot monitor the real-time distribution or diffusion of therapeutic agents within the muscle tissue. An important advancement in enhancing injection precision is the use of ultrasound (US) guidance as a complementary tool to EMG during botulinum toxin type A injections for CD, particularly for targeting deep cervical muscles such as the obliquus capitis inferior and longus colli. Furthermore, US guidance enables real-time visualization of the distribution and diffusion of the therapeutic agent within the muscle tissue (73, 100, 101). Additionally, a predictive model for individualized dosing based on electrophysiological parameters has yet to be established, and treatment decisions largely remain reliant on operator experience.

A key priority for future research is to establish clinical consensus and standardized guidelines that clearly define the diagnostic criteria for abnormal burst potentials, alterations in self-spectrum peaks, and quantitative thresholds for parameters such as the number of turns and amplitude. These criteria are essential for differentiating dystonic muscles from compensatory muscles and for correlating electrophysiological findings with the dosage of botulinum toxin injection to achieve precise, quantitatively informed treatment. In addition, techniques such as intermuscular coherence analysis should be further developed to localize abnormal central driving sources and construct a unique neural-muscular characteristic map of CD. Prospectively, computational modeling—such as “cortex-basal ganglia-muscle” circuit models to simulate pathological oscillations or pharmacokinetic-pharmacodynamic models integrated with individual EMG features—holds promise for deciphering mechanisms and personalizing botulinum toxin dosing. For DBS therapy, EMG should evolve into a core component for optimizing closed-loop, adaptive systems by providing real-time biomarkers (e.g., 4–12 Hz oscillatory power) to dynamically adjust stimulation parameters. Clarifying the abnormal patterns of frequency band oscillations in different motor tasks and comparing them with EMG changes during intracranial lesions will help elucidate the pathophysiological mechanism of CD at the circuit level. Technological innovation and clinical translation will also play important roles. The application of HD-sEMG to map the MEZs of cervical muscles can provide more comprehensive anatomical guidance to support injection techniques. Ultimately, the integration of machine learning with multimodal imaging is expected to advance the diagnosis and treatment toward a new era of automated, individualized, and precise medical care.

## 6 Conclusion

This review systematically delineates the integral role and recent advances of EMG in the comprehensive management of CD, spanning diagnosis, treatment, and pathophysiological research. As a pivotal neuroelectrophysiological tool, EMG contributes to differential diagnosis not only by assessing fundamental motor unit action potential characteristics—such as morphology and recruitment patterns—but also by identifying disease-specific signatures, including pathological co-contractions, aberrant oscillatory activities (e.g., 4–7 Hz coherence), and spectral alterations (e.g., attenuation of the physiological 8–14 Hz peak). These features provide objective evidence that aids in distinguishing CD from pseudodystonia and other hyperkinetic disorders such as tremor, myoclonus, tics, and chorea. Therapeutically, EMG guides precision botulinum toxin injection by localizing hyperactive muscles and motor endplate zones, while its quantitative parameters inform individualized dosing; moreover, it delivers real-time physiological feedback for target verification, intraoperative safety monitoring, and programming optimization in DBS. From a research perspective, EMG-derived biomarkers—such as corticomuscular coherence and central oscillatory drives—offer direct neurophysiological insights into the dysfunctional corticobasal ganglia-thalamic-brainstem circuits underlying CD. These applications underscore EMG as a cornerstone technology that supports the clinical workflow and continues to evolve toward more standardized, quantitative, and personalized patient management.
